# Psychological distance towards COVID-19: Geographical and hypothetical distance predict attitudes and mediate knowledge

**DOI:** 10.1007/s12144-021-02415-x

**Published:** 2021-10-31

**Authors:** Simon Blauza, Benedikt Heuckmann, Kerstin Kremer, Alexander Georg Büssing

**Affiliations:** 1 Institute for Science Education, Leibniz University Hannover, Am Kleinen Felde 30, Hannover, Germany; 2 Justus Liebig University, Institute for Biology Education, Karl-Glöckner-Straße 21C, 35394 Gießen, Germany

**Keywords:** COVID-19, Psychological distance, Behavior, Attitudes, Knowledge, Warning-app

## Abstract

While different antecedents have been examined to explain peoples’ reactions towards COVID-19, there is only scarce understanding about the role of the subjective closeness and distance to the pandemic. Within the current study, we applied the concept of psychological distance to understand the distance towards COVID-19 and investigated its (1) connection with preventive attitudes and proactive behaviors, (2) context-specific antecedents, and its (3) mediating effect of knowledge on attitudes. Using an online sample from a German quantitative cross-sectional study (*N* = 395, *M* = 32.2 years, *SD* = 13.9 years, 64.3% female) in July 2020, a time with a general low incidence of people infected with Sars-CoV2, we measured relevant socio-psychological constructs addressing COVID-19 and included further information from external sources. Based on a path model, we found geographical distance as a significant predictor of cognitive attitudes towards COVID-19. Furthermore, hypothetical distance (i.e., feeling to be likely affected by COVID-19) predicted not only participants’ affective, cognitive, and behavioral attitudes, but also the installation of a corona warning-app. While several variables affected the different dimensions of psychological distance, hypothetical and geographical distance mediated the effect of knowledge on attitudes. These results underline the role of geographical and hypothetical distance for health-related behaviors and education. For example, people will only comply with preventive measures if they feel geographically concerned by the disease, which is particularly challenging for fast-spreading global diseases such as COVID-19. Therefore, there is a need to clearly communicate the personal risks of diseases and address peoples’ hypothetical distance.

**Supplementary Information:**

The online version contains supplementary material available at 10.1007/s12144-021-02415-x.

## Introduction

Since the beginning of the COVID-19 pandemic, global efforts started to halt or delay the progression of pandemic by applying protective regulations. For example, the German government issued several measures to contain the virus outbreak, which partly still remained after the number of vaccinations was rising (Bundesregierung, 2020). The utilized measures included the temporal closing of borders, restriction of large parts of the public and social life, requirements of wearing mouth-and-nose covers, and social distancing. However, the adherence to these legislations depends on psychological reactions to the overall situation (Arden et al., 2020). To understand these reactions, it is of vital interest to investigate peoples’ attitudes and motivations to follow the recommended preventive behaviors such as wearing masks, practicing social distancing, or installing a contact tracing app for better tracking active cases (Arden et al., 2020).

The identification of variables relevant for preventive behaviors requires the consideration of different socio-psychological and demographic variables. Prior studies in the context of COVID-19 already investigated factors such as social support, media exposure, risk perception, or emotion regulation (Jaspal & Breakwell, 2021; Lin et al., 2020; Rubaltelli et al., 2020). In addition, Marinthe et al. (2020) showed that the motivation for protecting oneself may be connected to complying with specific containment measures. For demographic variables, studies demonstrated how age and prior illness may be connected to a larger concern about this issue (Lauri Korajlija & Jokic-Begic, 2020). These findings allow for a detailed intra-psychological view on peoples’ motivations. However, they may be biased by the fact, that the severity of the COVID-19 pandemic varies across geographical regions, which needs to be addressed as a relevant demographic characteristic (Ascani et al., 2020). While the geographical spread also has been investigated in prior studies (e.g. Ascani et al., 2020), it may be likely to assume that effects of the geographically spread on preventive behaviors are psychologically constructed. Currently, this psychological construction of distance to COVID-19 is scarcely understood. Besides explaining peoples’ adherence to the protective measures within the selected issue, the connection to health contexts such as COVID-19 may be relevant for general health-related decision-making.

For this health-related decision-making, knowledge represents a central reason for people to behave in concordance with health-favorable behaviors (Arnold, 2018). Therefore, science communication plays a major role for bringing scientific knowledge to action and may help people to better understand infection diseases such as COVID-19 (Bavel et al., 2020). But information alone may not lead to intended changes in behavior, which may also be affected by mediating variables such as attitudes or personal connections (Puspitasari et al., 2020). To sufficiently respond, a further investigation of possible mediators between knowledge and related constructs is essential.

The present paper addresses these two research gaps by applying the concept of psychological distance to the recent COVID-19 pandemic. The framework of psychological distance from Liberman and Trope (2008) describes peoples’ evaluation of distance to specific objects in the four dimensions of geographical, social, temporal, and hypothetical distance (see also Liberman & Trope, 2014). Within the study, we (1) investigated the connections of these dimensions with attitudes towards COVID-19 and the installation of the corona warning-app as an exemplary behavioral outcome. We then (2) tested possible antecedents of psychological distance and its (3) ability to mediate the link between knowledge and attitudes. Before describing the methods of our study, we explicate the tested hypotheses.

## Theoretical Background

### Psychological Distance as Closeness to COVID-19

*Psychological distance* describes the subjectively perceived distance to certain objects, events, or actions (Liberman & Trope, 2008; McDonald et al., 2015) and is characterized by four dimensions: geographical, temporal, social, and hypothetical distance (Liberman & Trope, 2014). Consequently, as described by Liberman and Trope (2008), people perceive issues as psychologically close, if the issue affects them in their direct spatial environment (geographical), in an immediate time frame (temporal), them personally (social), and if they evaluate to be likely concerned by the event at all (hypothetical).

Psychological distance is evaluated based on *construal level theory*, according to which humans use mental constructs of different abstraction levels to access objects (Trope & Liberman, 2010). Based on Liberman and Trope (2010), low-level and high-level construal may be differentiated from each other. Low-level construal is more concrete and detailed, as it is formed for rather close objects and events, whereas high-level construal, on the other hand, is more abstract and refers to distant objects and events (Liberman & Trope, 2010). Table 1 gives an overview of how the dimensions of psychological distance can be applied to the COVID-19 pandemic with the respective construal level.

**Table 1:** The four dimensions of psychological distance with the subsequent level of construal exemplified for the COVID-19 pandemic

Dimension	Continuum of construal		
	Low-level construal (concrete)		High-level construal (abstract)
Geographical distance	The COVID-19 pandemic affects my hometown.	The COVID-19 pandemic affects my home country.	The COVID-19 pandemic affects rather distant countries.
Temporal distance	The COVID-19 pandemic currently affects me.	The COVID-19 pandemic will still affect me in five years.	The COVID-19 pandemic will affect me for many years to come.
Social distance	The COVID-19 pandemic affects mainly people like me.	The COVID-19 pandemic mainly affects my family and friends.	The COVID-19 pandemic mainly affects other people.
Hypothetical distance	The COVID-19 pandemic will most likely affect me.	The COVID-19 pandemic is questionable to affect me.	The COVID-19 pandemic is unlikely to affect me.

The concept of psychological distance has already been applied to various health contexts, including virus-induced Ebola hemorrhagic fever or Zika disease (Johnson, 2018; Van Lent et al., 2017). In these studies, lower psychological distance was associated with an increased motivation for engaging in protective measures (i.e., avoiding traveling in Zika-effected areas). In another study, White et al. (2014) showed that people evaluate psychologically close viral diseases as more dangerous than psychologically distant ones. Besides this, psychological proximity increased the willingness for conforming with protective behaviors (i.e., paying for vaccines; White et al., 2014). However, it remains unknown whether such findings also hold true for psychological distance and COVID-19. Zheng et al. (2020) already applied the construct of psychological distance to COVID-19, but had a focus on general health and life satisfaction. While they found the regional number of confirmed COVID-19 cases as a negative predictor of the psychological distance towards the disease (Zheng et al., 2020), further possible correlates remain unknown. But a deeper understanding of these correlates may be important to understand peoples’ evaluation of the disease and their adherence to respective regulations.

### Attitudinal and Behavioral Correlates of Psychological Distance

*Attitudes* are defined as psychological tendencies towards specific objects, actions, ideas, behaviors, or persons (Ajzen, 1991). Psychological tendencies are expressed by the fact that a particular attitude object is evaluated with a certain degree of favor or disfavor. Thus, attitudes are understood as an overall evaluation of that object (Maio & Haddock, 2010). Eagly and Chaiken (1993) described attitudes as a tripartite construct, differentiating between affective, cognitive, and behavioral attitudes (also termed ABC model of attitudes; see also Rosenberg et al., 1960). While the cognitive component includes the beliefs about the attitude object, the affective component develops from associated emotions, and the behavioral dimension represents behavioral intentions towards the respective outcome (Ajzen, 2002; Rosenberg et al., 1960).

Prior studies already showed how attitudes are relevant for following protective behaviors such as washing hands and practicing social distancing in the context of COVID-19 (Lin et al., 2020). We therefore hypothesize the same relation in our study (H_1_). To investigate the effect of psychological distance on a specific behavioral outcome, we included the installation of a contact tracing app for mobile devices (H_2_). Similar to other countries, German authorities decided to develop such an application for mobile devices (“corona warning-app”), which may be used to monitor and improve the understanding of contact chains. As the app potentially helps to contain the spread of COVID-19 through better contact tracing (Blasimme & Vayena, 2020), it can be considered as a relevant protective behavior.

#### Hypothesis 1 (H_1_)

Psychological distance is connected to peoples’ affective, behavioral, and cognitive attitudes.

#### Hypothesis 2 (H_2_)

Psychological distance is connected to peoples’ willingness to install the corona warning-app.

### Antecedents of Psychological Distance towards COVID-19

People may strongly differ for their individual psychological distance. Based on prior studies, we hypothesized overall five different characteristics, that may be connected to peoples’ evaluation of psychological distance towards COVID-19. First of all, this includes the residence of people, as more cases were found in cities than on the countryside (H_3_; Schaff, 2020) and cases in specific districts (H_4_; Zheng et al., 2020). Additionally, cases in peoples’ social surrounding may be one of the few unmediated experiences of the pandemic, which is why they also may affect the psychological distance (H_5_). Nguyen et al. (2020) further showed that the profession is a relevant demographical variable that may be connected to the perception of COVID-19 (H_6_; Nguyen et al., 2020). In particular, the daily contact with COVID-19 patients is an extraordinary situation for medical personnel (Bielicki et al., 2020). Finally, we assumed that more knowledge (e.g., about ways of spreading the virus, about understanding the research, etc.) has an influence on the psychological distance (H_7_). This would be in line with construal-level theory, which assumes less psychological distance for issues with more concrete representations (i.e., more knowledge; Liberman & Trope, 2014) and was also found in prior studies (Büssing et al., 2021).

#### Hypothesis 3 (H_3_)

People from cities report a smaller psychological distance towards COVID-19 than people from the countryside.

#### Hypothesis 4 (H_4_)

People with more cases in their district report a smaller psychological distance towards COVID-19.

#### Hypothesis 5 (H_5_)

People that report cases of COVID-19 in their social environment report a smaller psychological distance towards COVID-19.

#### Hypothesis 6 (H_6_)

People that work in the medical sector report a smaller psychological distance towards COVID-19 than people from other professions.

#### Hypothesis 7 (H_7_)

People with more knowledge about COVID-19 report a smaller psychological distance towards COVID-19.

Furthermore, we controlled for the demographic variables of gender, age, and the level of education, which have been found to be connected to concern of COVID-19 in prior studies (Lauri Korajlija & Jokic-Begic, 2020).

### Mediation of Knowledge and Attitudes

Variables such as knowledge may be seen as a requirement for understanding governmental regulations surrounding COVID-19 and may lay the foundation for a positive evaluation of the taken actions (Hamza et al., 2020). But like other diseases, this connection may be affected by other factors. Given the hypothesized connections of psychological distance and the overall evaluation, we finally investigated, if psychological distance acts as a mediator of the effect between knowledge and attitudes (H_8_). Insights into such a mediating effect would be relevant for educational interventions aiming for increasing attitudes about COVID, as the success of such interventions may depend on peoples’ individual psychological distance.

#### Hypothesis 8 (H_8_)

Psychological distance mediates the effect of knowledge on attitudes.

## Methods

### Research Design and Sample

To investigate the directed hypotheses, we chose a cross-sectional quantitative research design with a standardized online questionnaire, which was distributed via the platform *SociSurvey* (https://www.soscisurvey.de/). The survey was online for two weeks from July 1st 2020 to July 15th 2020, in direct proximity to the publication of the corona warning-app. The link to the questionnaire was distributed via convenience sampling, mainly through social networks (e.g. Facebook, Instagram, Snapchat, Twitter and WhatsApp). Participants needed on average approximately 11 min to complete the questionnaire. While the questionnaire was distributed in German, the final version of all items with corresponding English translations can be found in the Supplementary Table 1.

Inclusion criteria for study participants were a minimum age of 18 years and a place of residence in Germany (self-reported data). Overall, a total of 395 persons completed the survey (mean_age_ = 32.22 years, SD_age_ = 13.86 years, age range from 18 to 80 years, 64.3% female,). Most of the participants had at least a university entrance qualification (“Abitur”, *n* = 329, 83.3%). In addition, about half of the participants lived in urban or suburban areas (*n* = 195, 49.4%). Based on the self-reported professions, we have divided the participants into certain occupational groups that are probably more affected by COVID-19. These groups were education (n = 123, 31.3%), medical (*n* = 32, 8.1%), and other (*n* = 157, 39.7%). Given these demographics, further studies could further generalize the results to other more general samples.

### Questionnaire Design

#### Demographic and Other Contextual Variables

While age, profession, and district were assessed in an open-ended format, gender (male, female, diverse), educational level, installation of the corona warning-app, and residence (rural to urban) were formulated as closed questions. The district (german “Landkreis”) was used to determine the number of active cases with the help of the “Robert Koch-Institute: COVID-19-Dashboard” (Esri Deutschland GmbH, 2020). We also asked, if participants knew about a positively tested COVID-19 case in their close social surrounding and whether they have the corona warning-app installed with closed binary items.

#### Psychological Distance

Due to the lack of standardized scales to measure psychological distance, we adapted the scale based on existing studies (Büssing et al., 2020; Jones et al., 2017). We designed three items for each of the four dimensions of psychological distance as displayed in Table 1. These items were constructed as statements, which allow people to indicate their agreement with the respective items (Bryman, 2008). This resulted in a total of 12 items, which were assessed on a 6-point rating scale. Since several items were measured as concern and not as distance, these items have been reversely coded as displayed in the supplemental material (Supplemental Table 2) before combining all items of the respective dimension to one mean value (Trope & Liberman, 2010).

#### Attitudes

Based on the tripartite model of attitudes by Eagly and Chaiken (1993), two positively and two negatively formulated items were designed for each of the affective, cognitive, and behavioral dimension of attitudes. This resulted in a total of 12 items for the measurement of attitudes and each item was assessed on a 6-point rating scale. Mean values for the individual items were formed to create scales for the different dimensions. The negatively formulated items were reversely coded before they were aggregated for the analysis.

#### Knowledge Test

The study included a knowledge test on COVID-19 consisting of seven items, which was adapted from other studies (Hamza et al., 2020). The knowledge tested was about risk groups, symptoms, statistical indicators of the pandemic, the first occurrence of the virus, the use of mouth-nose covers, and the molecular mechanism of the virus’ entry into the human body. The items were presented in a closed answer format. Correct answers were scored with 1 and the number of correct responses was summed to a knowledge score, ranging from 0 to 7. The higher the score, the higher the knowledge about COVID-19.

### Statistical Analysis

Before investigating the research questions, we ensured the measurement abilities of the scales and conducted factor analyses for the psychological distance (Supplemental Table 2) and attitudes (Supplemental Table 3). As recommended by Schermelleh-Engel et al. (2003), different indices were used to evaluate the fit of the model to the data (chi-square values (χ^2^), root mean square error of approximation (RMSEA), standardized root mean square residual (SRMR), comparative fitting index (CFI)), which were then evaluated according to the cut-offs for good fit (χ^2^ ≤ 2, CFI ≥ 0.97, RMSEA ≤0.05, and SRMR ≤0.05). As the dimensions of psychological distance were correlated only with small to medium strength (0.14 < r < 0.52), we analyzed the research questions based on the individual dimensions.

We used a path model with the means of the respective variables to investigate the connection of psychological distance to the attitudinal and behavioral outcomes (H_1_ and H_2_). For model evaluation the same indices were used as for the confirmatory factor analysis. To test the antecedents, we used a two-step regression approach with the individual dimensions of psychological distance as dependent variables (H_3–7_). Finally, we estimated indirect effects of knowledge with individual dimensions of psychological distance as a mediator with mediator analyses based on four different models (H_8_).

We used robust estimators such as spearman-rho as a correlation coefficient, a robust maximum likelihood estimator for the path model, robust regression for the multiple regressions, and robust mediation tests (Field & Wilcox, 2017). All computations were made with R-Studio (version 1.3.1073). The data, code, and other materials for the replication of the analyses can be found in the supplemental material of the paper.

### Measurement Results of Psychological Distance

Based on the factor analysis, we obtained good measurement abilities for the psychological distance, even when slight modifications were needed (Supplementary Table 2). The adapted model achieved a satisfactory fit to the data (χ^2^ (21) = 46,478, CFI = 0.97, RMSEA = 0.06, SRMR = 0.04). To confirm the four dimensions of psychological distance, we estimated an alternative one-dimensional model, that showed the worst fit (χ^2^ (27) = 316.645, CFI = 0.67, RMSEA = 0.18, SRMR = 0.10). The modified theoretical model was significantly better in explaining the data than the modified one-dimensional (χ^2^ (6) = 238.6, *p* < 0.001).

The tests for the measurement abilities of the attitudes can be accessed in the supplementary material (Supplementary Table 3). The factor analyses confirmed in accordance with the Cronbach’s alpha the theoretical assumption of the tripartite model. All values, further descriptive statistics and bivariate correlations are displayed in Table 2.

**Table 2:** Bivariate correlations between the dependent variables (dimensions of psychological distance and all attitude components), bivariate correlations of dependent and independent variables (hypotheses and control variables), and descriptive statistics of dependent variables

	1	2	3	4	5	6	7	8
1. Geographical distance	–	.14	.23**	.52**	−.16	−.31**	−.24**	−.12
2. Temporal distance	.14**	–	.20*	.30**	−.03	−.09	−.15	−.06
3. Social distance	.23**	.20**	–	.37**	−.13	−.08	−.14	−.06
4. Hypothetical distance	.52**	.30**	.37**	–	−.21**	−.35**	−.31**	−.22**
5. Affective att.	−.16**	−.03	−.13*	−.21**	–	.39**	.30**	.19*
6. Cognitive att.	−.31**	−.09	−.08	−.35**	.39**	–	.44**	.26**
7. Behavioral attitudes	−.24**	−.15**	−.14	−.31**	.30**	.44**	–	.15
8. Corona warning-app	−.12*	−.06	−.06	−.22**	.19**	.26**	.15**	–
9. Residence	−.18**	.07	.03	−.11*	.00	.04	.02	.11*
10. Cases in district	−.15**	−.05	−.11*	−.14*	−.11*	.00	.01	.15**
11. Case in surrounding	.08	.00	.05	.12*	.07	.07	.04	.01
12. Medical profession	−.05	.03	−.18**	−.09	.04	.00	.02	.01
13. Knowledge	−.13*	−.07	.01	−.15**	.07	.13*	.12*	.15**
14. Gender	−.03	−.11*	−.07	−.03	−.04	.07	.25**	−.10
15. Age	.00	−.04	.05	.01	.07	.09	.02	−.09
16. Level of education	−.15**	.00	−.06	−.20**	.03	.12*	.11	.26**
Number of Items	2	2	2	3	4	3	4	1
Mean	2.53	3.56	3.98	2.57	3.45	4.96	4.90	.55
Standard deviation	1.05	1.18	1.09	1.08	1.08	1.01	0.85	.50
Median	2.50	3.50	4.00	2.33	3.50	5.12	5.00	–
Skewness	0.55	0.02	−0.29	0.58	−0.15	−1.55	−1.11	−.20
Kurtosis	0.10	−0.58	−0.10	−0.11	−0.44	2.76	1.85	−1.97
Cronbach’s α	.58	.85	.55	.76	.70	.85	.70	–

Correlations in the upper half of the correlation matrix are adjusted for multiple tests. * = *p* < .05, ** = *p* < .01, Corona warning-app was coded (0) not installed and (1) installed, Gender was coded as (1) male and (2) female, Cases in social surrounding was coded (1) yes and (2) no, medical profession was coded (0) not working in a medical profession and (1) working in a medical profession, Residence was coded from (1) rural to (5) urban

## Results

### Effects of Psychological Distance on Behavioral Correlates

**Fig. 1:**
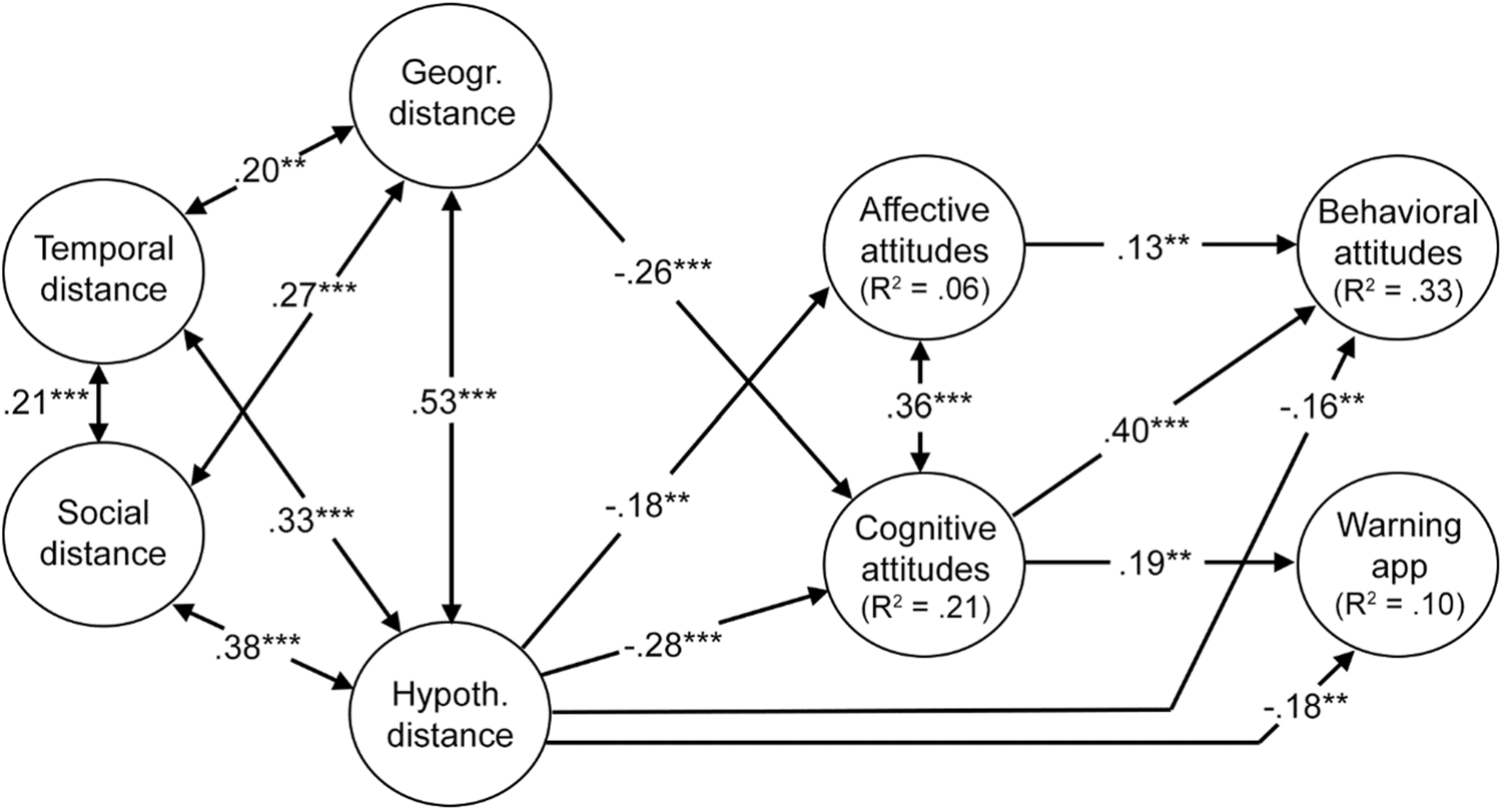
Final path model for the prediction of the behavioral component of attitudes and installation of corona warning-app by psychological distance and other selected study variables

The path model as displayed in Fig. 1 showed a very good fit to the data (χ^2^ (28) = 578.331, CFI = 1.00, RMSEA = 0.00, SRMR = 0.00). While the geographical distance towards COVID-19 was a significant predictor only for the cognitive attitudes (*β* = −0.26, *p* < 0.001), hypothetical distance was a significant predictor for the affective (*β* = −0.18, *p* < 0.01), cognitive (*β* = −0.28, *p* < 0.001), and the behavioral attitudes (*β* = −0.16, *p* < 0.01). Finally, hypothetical distance also significantly predicted the installation of the corona warning-app (*β* = −0.18, *p* < 0.01). The detailed effects and 95% confidence intervals can be found in the Supplementary Table 4.

The path model explained 33% of the variance for the behavioral attitudes (adj. *R*^*2*^ = 0.33), 21% of the variance for the cognitive attitudes (adj. *R*^*2*^ = 0.21), but only 10% of the variance for the installation of the corona warning-app (adj. *R*^*2*^ = 0.10) and 6% of the variance for affective attitudes (adj. *R*^*2*^ = 0.06).

### Antecedents of Psychological Distance

The results for the antecedents of the individual dimensions of psychological distance are described in Table 3. In the first regression step, the geographical distance was negatively predicted by the residence (*β* = −0.17, *p* < 0.01) and knowledge (*β* = −0.14, *p* < 0.05). Cases in the district, which showed a bivariate correlation to the geographical distance (*r* = −0.15, *p* < 0.01; Table 2), was no significant predictor (*β* = −0.03, *p* > 0.05). Concerning the temporal distance, none of the variables showed a significant relationship. The social dimension was significantly predicted by the medical profession (*β* = −0.24, *p* < 0.01) and cases in the district (*β* = −0.10, *p* < 0.01). Finally, the hypothetical distance was significantly predicted by the cases in the social surrounding (*β* = 0.12, *p* < 0.01) and knowledge (*β* = −0.19, *p* < 0.01).

**Table 3:** Regression results of the antecedents for the dimensions of the psychological distance with standardized regression coefficients (β) and standard error (SE)

Predictors	Psychological distance			
	Geographical	Temporal	Social	Hypothetical
Model 1: Hypotheses				
Intercept	2.47*** (.05)	3.57*** (.06)	3.99*** (.06)	2.51*** (.06)
Residence	−.17** (.06)	.08 (.06)	.04 (.06)	−.11 (.06)
Cases in district	−.03 (.04)	.01 (.03)	−.10** (.03)	.02 (.04)
Case in social surrounding	.07 (.05)	.00 (.06)	.04 (.06)	.12* (.06)
Medical sector	−.06 (.07)	.05 (.09)	−.24** (.07)	−.09 (.05)
Knowledge	−.14* (.06)	−.06 (.07)	.01 (.05)	−.19** (.06)
R^2^ (Adjusted R^2^)	.06 (.05)	.00 (.00)	.05 (.04)	.06 (.05)
Model 2: Hypotheses and demographic control variables				
Intercept	2.47*** (.05)	3.57*** (.06)	3.99*** (.06)	2.51*** (.05)
Residence	−.16** (.06)	.07 (.06)	.07 (.06)	−.08 (.06)
Cases in district	−.02 (.04)	.02 (.03)	−.10** (.03)	.03 (.04)
Case in social surrounding	.06 (.05)	.00 (.06)	.04 (.06)	−.11 (.06)
Medical sector	−.06 (.07)	.06 (.09)	−.24** (.07)	−.09 (.05)
Knowledge	−.13* (.06)	−.05 (.08)	.02 (.05)	−.18** (.06)
Gender	.00 (.05)	−.14 (.06)	−.06 (.06)	−.02 (.06)
Age	−.03 (.07)	−.06 (.07)	.10 (.06)	−.07 (.06)
Level of education	−.10 (.07)	−.05 (.06)	−.04 (.06)	−.20** (.07)
R^2^ (Adjusted R^2^)	.07 (.05)	.02 (.00)	.07 (.05)	.09 (.07)

*F* F-statistic, *df* Degrees of freedom, *R*^*2*^ Explained variance, * = *p* < 0.05, ** = *p* < 0.01, *** = *p* < 0.001, Gender was coded as (1) male and (2) female, Cases in social surrounding was coded (1) yes and (2) no, medical profession was coded (0) not working in a medical profession and (1) working in a medical profession, Residence was coded from (1) rural to (5) urban

The demographic control variables, which were added in the second regression step, had only a small impact on the results of the regression analyses. All effects remained similar with small decreases, except for the hypothetical distance. In the second step this dependent variable was only predicted by level of education (*β* = 0.20, *p* < 0.01) and knowledge (*β* = −0.18, *p* < 0.01), but not by cases in the social surrounding (*β* = −0.11, *p* > 0.05). Overall, the models explained only a small amount of variance for the individual dimensions of psychological distance, as no model explained more than 10% of the variance in the dependent variables (0.00 < *R*^*2*^ < 0.09).

### Mediation of Knowledge

As displayed in Table 4, geographical and hypothetical distance mediated the effect of knowledge on cognitive and behavioral attitudes. While knowledge was a predictor for the hypothetical (*β* = 0.21, *p* < 0.001) and geographical distance (*β* = 0.19, *p* < 0.001), hypothetical distance fully mediated the effect of knowledge on cognitive attitudes (*β*_TOTAL_ = 0.11, *p* < 0.01), as knowledge itself had no direct effect on cognitive attitudes (*β*_DIRECT_ = 0.07, *p* > 0.05). The same full mediation was found for the behavioral attitudes (*β*_TOTAL_ = 0.10, *p* < 0.05). While geographical distance partially mediated the effect of knowledge on the cognitive attitudes (*β*_TOTAL_ = 0.14, *p* < 0.01), as knowledge also was a direct predictor (*β*_DIRECT_ = 0.10, *p* < 0.05), it again fully mediated the effect of knowledge on the behavioral attitudes (*β*_TOTAL_ = 0.09, *p* < 0.05).

**Table 4:** Mediation analyses of the indirect effects between knowledge and the cognitive and behavioral attitudes with hypothetical (model 1–2) and geographical distance (model 3–4) as mediators

Model 1	Hypothetical distance			Cognitive attitudes		
	*β* (SE)	LL	UL	*β* (SE)	LL	UL
Hypothetical distance	–	–	–	−0.20*** (0.05)	−0.29	−0.11
Knowledge (direct)	−0.21*** (0.06)	−0.33	−0.08	0.07^n.s.^ (0.04)	−0.01	0.15
Knowledge (indirect)	–	–	–	0.04*** (−)	0.02	0.08
Knowledge (total)	–	–	–	0.11** (0.04)	0.03	0.20
R^2^ (adj. R^2^)	0.04 (0.03)			0.10 (0.10)		
Model 2	Hypothetical distance			Behavioral attitudes		
	*β* (SE)	LL	UL	*β* (SE)	LL	UL
Hypothetical distance	–	–	–	−0.22*** (0.05)	−0.32	−0.13
Knowledge (direct)	−0.21*** (0.06)	−0.33	−0.08	0.05^n.s.^ (0.04)	−0.03	0.13
Knowledge (indirect)	–	–	–	0.05*** (−)	0.02	0.09
Knowledge (total)	–	–	–	0.10* (0.04)	0.01	0.18
R^2^ (adj. R^2^)	0.04 (0.03)			0.10 (0.10)		
Model 3	Geographical distance			Cognitive attitudes		
	*β* (SE)	LL	UL	*β* (SE)	LL	UL
Geographical distance	–	–	–	−0.18** (0.06)	−0.29	−0.07
Knowledge (direct)	−0.19*** (0.06)	−0.30	−0.08	0.10* (0.04)	0.02	0.18
Knowledge (indirect)	–	–	–	0.03** (−)	0.01	0.07
Knowledge (total)	–	–	–	0.14** (0.04)	0.05	0.22
R^2^ (adj. R^2^)	0.03 (0.03)			0.08 (0.08)		
Model 4	Geographical distance			Behavioral attitudes		
	*β* (SE)	LL	UL	*β* (SE)	LL	UL
Geographical distance	–	–	–	−0.18*** (0.05)	−0.27	−0.08
Knowledge (direct)	−0.19*** (0.06)	−0.30	−0.08	0.06^n.s.^ (0.04)	−0.02	0.14
Knowledge (indirect)	–	–	–	0.03*** (−)	0.01	0.07
Knowledge (total)	–	–	–	0.09* (0.04)	0.01	0.17
R^2^ (adj. R^2^)	0.03 (0.03)			0.06 (0.06)		

*β* = Standardized regression coefficient, SE = Standard error, LL = Lower limit of the 95% confidence interval, UL = Upper limit of the 95% confidence interval, ^n.s.^ = not significant, * = *p* < 0.05, ** = *p* < 0.01, *** = *p* < 0.001, *R*^*2*^ Explained variance, *adj. R*^*2*^ Adjusted explained variance

## Discussion

### Behavioral Correlates

We found a significant relationship between the geographical distance and the cognitive attitudes towards COVID-19 (H_1_). This implies differences in the cognitive evaluation of the pandemic, depending on the experienced geographical distance. This result was in line with prior studies, since spatial proximity has been found as prerequisite for psychological distance (Arden et al., 2020). Furthermore, this underlines the priority of sufficient science communication, as a disease momentarily far away may quickly spread into geographical closeness, which also has been shown for COVID-19 (Whitworth, 2020). But besides geographical distance, especially the hypothetical distance showed to be an antecedent of the subsequent attitudes.

Hypothetical distance predicted all attitudes, which is in line with prior studies. For example, Lin et al. (2020) found, that people with increased risk perception and corresponding self-efficacy are more likely to take preventive measures to contain COVID-19 in order to minimize their own risk. This may be explainable, for example, by the higher risk people may attribute to the disease if they feel to be likely affected by COVID-19. Another study showed, how people believing in conspiracy theories are unlikely to follow preventive measures, but may be more motivated if they experience a risk of death for themselves (Marinthe et al., 2020). Higher skepticism of COVID-19 is also connected to a smaller perceived likelihood of people in close distance dying due to the virus (Latkin et al., 2021). This could be explained by a change in hypothetical distance, which may induce a change of abstract representations of COVID-19 to more concrete ones. Such a concretization could be the objective of formal and informal educational activities, which may be used to communicate with people, for example through social media.

Furthermore, hypothetical distance significantly predicted the installation of the corona warning-app. People therefore more likely installed the app, if they believed that they will be concerned by COVID-19 (H_2_). The geographical distance showed a bivariate correlation to the installation of the corona warning-app, but was no significant predictor in the path model. This underlines the role of hypothetical distance as an antecedent of protective behaviors in the recent COVID-19 pandemic. Therefore, a more concrete communication about personal risks may be able to foster people’s motivation to install the corona warning-app, if governments or institutions plan to implement this containment measure.

Regardless of the significant effect of hypothetical distance, the predictors were able to explain only about 10 % of the variance in the dependent variable of installing the corona warning-app. This was a much smaller amount than was explained for the behavioral attitudes, regardless of the conceptual similarities between both variables. This may partly be explained by the binary coding of the variable. For example, some participants may have had the willingness to install the app, but were not able to do so, for example due to owning a too old smartphone. This variance could not be explained and therefore may have entailed a larger error variance. Even when the models are unable to explain this error variance, the effect of the hypothetical distance makes also sense for this behavioral outcome. To explain more variance, it would be possible to rely on a Likert scale measurement in further studies, similar to prior studies (Altmann et al., 2020).

### Antecedents of Psychological Distance

Our results showed different antecedents for the individual dimensions of psychological distance. Concerning our third hypothesis (H_3_), residence significantly predicted only the geographical distance. This illustrates how the higher number of cases in cities also leads to a smaller geographical distance in these densely populated places. This is in line with prior research, which showed that people from rural areas may hold more negative attitudes towards COVID-19 (Chen & Chen, 2020). The result may be explainable due to the different challenges people are facing. For example, people living in the city probably have more frequent contact with strangers (e.g., when using public transport) than people from the countryside. For containing the disease, this implicates that different communication and education strategies may be taken in cities than on the countryside, due to the differing concern of the issue in these districts.

The cases in the district (H_4_) were only predictive for the social dimension of psychological distance. While the social distance was neither related to the attitudinal nor the behavioral outcomes, the effect of cases in the district only on this dimension contradicts prior expectations. As we found a correlation between the geographical distance and cases in the district, a similar connection was expected in the regression. Nonetheless, other variables were better in explaining the variance in the dependent variable. One reason for this could be the connection of residence and cases in the district, as cities generally have also more cases (Schaff, 2020). Another reason may be the time of the study. The questionnaire was filled in July 2020, a time, in which only some districts already had a lot of cases. This could have been affected the results. For future studies a more comparative approach between times with high and low cases in general would be interesting, for example also in the longitudinal perspective. This also concerns the next variable, the cases in the direct surrounding of the participants.

The cases in social surrounding (H_5_) were significant predictors of the hypothetical distance. This is a very interesting result, which illustrates how a more concrete experience of the pandemic (i.e., knowing someone infected) entails a more concrete representation of the pandemic. This result is in line with construal level theory (Liberman & Trope, 2008). Nonetheless, we found this connection only in the first step of our regression. In the second step, this variable had no longer a significant relationship to the hypothetical distance, which was now predicted by the level of education. As the participants were rather well educated, future studies need to replicate this with more diverse samples.

Participants’ profession only affected the social dimension, for which people from the medical sector reported a smaller distance (H_6_). This is in line with prior studies, which showed how people in medical professions are tested for COVID-19 more often than people in other professions (Allen et al., 2020). They also found that medical workers are at increased risk of infection and should be given greater priority for testing. This could be a reason for a lower psychological distance in these professions, but further studies could investigate in how far COVID testing may affect psychological distance.

Concerning our final hypothesis (H_7_), we identified knowledge as the only antecedent, which predicted more than one dependent variable, as knowledge predicted the geographical as well as the hypothetical distance. This, again, confirms the assumptions of construal-level-theory about how a smaller distance entails more concrete representations about the respective issue (Trope & Liberman, 2010) and is in line with prior studies (Büssing et al., 2021). The fact that more knowledge leads to less geographical distance may be since people with more knowledge are also informed about the current pandemic situation in their district.

While this underlines the role of good public education about the pandemic, we found level of education as the only demographic variable that predicted psychological distance. This is in line with prior research (Cvetković et al., 2020), but also contradicts prior expectations. For example, it would have been reasonable to expect a connection between age and psychological distance, given the higher risk of a bad course for older age groups. In our study, only 15% of the participants were older than 50. This may be due to the nature of the study, which was established as an online study. Future studies could also further generalize this to explicitly selected older age groups.

### Mediation of Knowledge and Attitudes

Our results showed geographical and hypothetical distance as mediators of the effect of knowledge on attitudes, in line with our hypothesis (H_8_). Due to the full mediation of psychological distance for some models, attempts of informing the public with more knowledge need to consider the perceived distance to the issue. While prior studies found severe differences between peoples’ knowledge concerning COVID-19 (Hamza et al., 2020), knowledge will only then entail more positive attitudes, if people experience a small geographical or hypothetical distance.

This mediating role could also be a vital key for understanding how people may come to different conclusions with the same rational information about COVID-19. Therefore, future studies should more explicitly investigate this connection, as the missing hypothetical distance may be one key factor for people disbelieving the governmental information, besides other personality related factors (Alper et al., 2020; Marinthe et al., 2020).

A practical implication for formal and informal education could be to tailor materials in concordance with the specific geographical situation of infections (Büssing & Heuckmann, 2021). In districts with only few infections, there should be a clear focus on communicating the risk for exponential growth of a fast-spreading disease like COVID-19, and thus paying attention to the people’s hypothetical distance towards the disease. In districts with more cases, the need to clarify the geographical risk of COVID-19 may not be as important as in regions without these cases. Despite these learnings for the current pandemic, different limitations need to be observed that may be addressed in future research.

### Limitations and Further Research Directions

Besides the already discussed limitations, we were unable to find suitable predictors for the temporal dimension of psychological distance. In part, this may also be due to the timepoint of the study, since the data was collected during a time of low incidence in Germany and Covid-19 outbreaks mainly took place in few specific districts. Besides this, all models generally explained only a small amount of the variance, even when this was similar in other studies (Zheng et al., 2020). The variance explained may partly be accounted to some error variance induced by the online study, as all people may completed the study in different and unstandardized ways, but the concordance to other studies may indicate the need for further work surrounding the construct of psychological distance. For this, also connections with other health-related variables such as the Big Five personality model could be relevant (Aboul-ata & Qonsua, 2021).

Regardless of these limitations, we were able to investigate the relevance of perceived affection with COVID-19. One of the differences to prior studies was the application of a multidimensional way of measuring psychological distance, in contrast to prior studies, which neglected the multiple dimensions of the construct (e.g. Zheng et al., 2020) or measured each dimension only with one item (Büssing et al., 2019). Based on this measurement approach, we were able to describe specific effects of hypothetical distance, which may be an objective for future studies. This could include for example the role of risk estimation and hypothetical distance, as a higher perceived risk may lead to a smaller hypothetical distance (Jaspal & Breakwell, 2021). Regardless of these desiderata, different implications already can be concluded from our study.

## Implications and Conclusion

The present study represents the foundation for understanding connections to health contexts such as COVID-19. Specifically, we were able to identify connections between geographical and hypothetical distance with cognitive, affective, and behavioral attitudes. As the cognitive evaluation is affected by psychological distance, the construal may also be relevant in further phases of the pandemic, for example, when vaccinations become available to large parts of the population. Particularly, some people may be demotivated to get vaccinated if the number of cases in their district or social surrounding is low. Besides this, the mediation of knowledge illustrated, how personality related variables affect peoples’ information processing, which is important for the way governments inform the population. Public information campaigns therefore also need to consider the role of geographical and hypothetical distance. The further consideration of such effects may also be able to deepen the understanding of health-related decision-making in general. For example, a high-level construal (i.e., a large psychological distance) of a disease may affect the overall decision-making and higher-order thinking may depend on a more detailed understanding of the issue, which may be only possible in combination with a concrete construal (i.e., small psychological distance).

As pointed out by these different implications, the present study was able to point out the role of psychological distance for the specific pandemic situation. Abstracted from this, the study may be able to lay ground for a deeper understanding of how people feel connected towards specific issues and how this may affect their behaviors.

## Supplementary Information

Below is the link to the electronic supplementary material.

English and German versions of the dependent variables (ESM 1) (PDF 73 kb)

Factor analysis of psychological distance (ESM 2) (PDF 76 kb)

Factor analysis of attitudes (ESM 3) (PDF 104 kb)

Detailed results for moderation analysis (ESM 4) (PDF 114 kb)

Dataset (ESM 5) (CSV 83 kb)

R-script for replication of the analysis (ESM 6) (R 8 kb)

## Data Availability

The data, materials, and code for the replication of all analyses is available in the supplemental material of the manuscript.
