# Supplementary material for: Psychological distance towards COVID-19: Geographical and hypothetical distance predict attitudes and mediate knowledge
Source: Curr Psychol. 2021 Oct 31;42(10):8632–43. doi: 10.1007/s12144-021-02415-x (PMC8557103; doi:10.1007/s12144-021-02415-x)
Supplement: Supplementary file 1 — English and German versions of the dependent variables (ESM 1) (PDF 73 kb) [file 12144_2021_2415_MOESM1_ESM.pdf]

Supplementary table 1
Overview of the English and German versions of all items of dependent variables.

Item	English	German
**Geographical distance**		
PDgeo_01	The COVID-19 pandemic affects my home town.	Die COVID-19-Pandemie betrifft meinen Wohnort.
PDgeo_02	The COVID-19 pandemic affects my home country.	Die COVID-19-Pandemie betrifft mein Heimatland.
PDgeo_03	The COVID-19 pandemic affects rather distant countries.	Die COVID-19-Pandemie betrifft eher weit entfernte Länder.
**Temporal distance**		
PDtemp_01	The COVID-19 pandemic currently affects me.	Die COVID-19-Pandemie betrifft mich gegenwärtig.
PDtemp_02	The COVID-19 pandemic will still affect me in five years.	Die COVID-19-Pandemie wird mich noch in fünf Jahren betreffen.
PDtemp_03	The COVID-19 pandemic will affect me for many years to come.	Die COVID-19-Pandemie wird mich noch in vielen Jahren betreffen.
**Social distance**		
PDsoci_01	The COVID-19 pandemic mainly affects people like me.	Die COVID-19-Pandemie betrifft vor allem Menschen wie mich.
PDsoci_02	The COVID-19 pandemic mainly affects my family and friends.	Die COVID-19-Pandemie betrifft vor allem meine Familie und Freunde.
PDsoci_03	The COVID-19 pandemic mainly affects other people.	Die COVID-19-Pandemie betrifft vor allem andere Menschen.
**Hypothetical distance**		
PDhypo_01	The COVID-19 pandemic will most likely affect me.	Die COVID-19-Pandemie betrifft mich sehr wahrscheinlich.
PDhypo_02	The COVID-19 pandemic is questionable to affect me.	Dass die COVID-19-Pandemie mich betrifft, halte ich für fraglich.
PDhypo_03	The COVID-19 pandemic is unlikely to affect me.	Dass die COVID-19-Pandemie mich betrifft, halte ich für unwahrscheinlich.
**Affective attitudes**		
att_pos_aff_01	The measures to contain COVID-19 imposed from March onward have made my life easier.	Die ab März verhängten Maßnahmen zur Eindämmung von COVID-19 haben mich erleichtert.
att_pos_aff_02	The measures to contain COVID-19 imposed from March onward have pleased me.	Die ab März verhängten Maßnahmen zur Eindämmung von COVID-19 haben mich erfreut.
att_neg_aff_01	The measures to contain COVID-19 imposed from March onward have dismayed me.	Die ab März verhängten Maßnahmen zur Eindämmung von COVID-19 haben mich bestürzt.
att_neg_aff_02	The measures to contain COVID-19 imposed from March onward have frustrated me.	Die ab März verhängten Maßnahmen zur Eindämmung von COVID-19 haben mich frustriert.

**Cognitive attitudes**		
att_pos_cog_01	The measures to contain COVID-19 imposed from March onwards were, I believe, appropriate.	Die ab März verhängten Maßnahmen zur Eindämmung von COVID-19 waren meiner Überzeugung nach angemessen.
att_pos_cog_02	The measures to contain COVID-19 imposed in March were, in my opinion, effective.	Die ab März verhängten Maßnahmen zur Eindämmung von COVID-19 waren meiner Meinung nach effektiv.
att_neg_cog_01	The measures to contain COVID-19 imposed in March were, in my opinion, disproportionate.	Die ab März verhängten Maßnahmen zur Eindämmung von COVID-19 waren meiner Überzeugung nach unverhältnismäßig.
att_neg_cog_02	The measures to contain COVID-19 imposed in March were, in my opinion, not far-reaching enough.	Die ab März verhängten Maßnahmen zur Eindämmung von COVID-19 waren meiner Überzeugung nach nicht weitreichend genug.
**Behavioral attitudes**		
att_pos_beh_01	The measures to contain COVID-19, which were imposed from March on, I followed.	Die ab März verhängten Maßnahmen zur Eindämmung von COVID-19 habe ich befolgt.
att_pos_beh_02	The measures to contain COVID-19, which were imposed from March on, have made me avoid the public.	Die ab März verhängten Maßnahmen zur Eindämmung von COVID-19 haben mich dazu bewegt, die Öffentlichkeit zu meiden.
att_neg_beh_01	The measures to contain COVID-19, which were imposed in March, I ignored.	Die ab März verhängten Maßnahmen zur Eindämmung von COVID-19 habe ich ignoriert.
att_neg_beh_02	The measures to contain COVID-19 imposed in March did not prevent me from going out into the public.	Die ab März verhängten Maßnahmen zur Eindämmung von COVID-19 haben mich nicht gehindert in die Öffentlichkeit zu gehen.
