# Supplementary material for: Psychological distance towards COVID-19: Geographical and hypothetical distance predict attitudes and mediate knowledge
Source: Curr Psychol. 2021 Oct 31;42(10):8632–43. doi: 10.1007/s12144-021-02415-x (PMC8557103; doi:10.1007/s12144-021-02415-x)
Supplement: Supplementary file 2 — Factor analysis of psychological distance (ESM 2) (PDF 76 kb) [file 12144_2021_2415_MOESM2_ESM.pdf]

Supplemental table 2

Factor loadings (λ) and fit indices of the confirmatory factor analyses (CFA) for measuring the psychological distance towards COVID-19 calculated with an initial, modified (four dimensional), and modified (one dimensional) factor structure.

Item	Factor loading		
	Initial	Modified (four dim.)	Modified (one dim.)
**Geographical**			
The COVID-19 pandemic affects my home town. (PDgeo_01)*	.65	.68	.53
The COVID-19 pandemic affects my home country. (PDgeo_02)*	.66	.63	.52
The COVID-19 pandemic affects rather distant countries. (PDgeo_03)	.42	-	-
**Temporal**			
The COVID-19 pandemic currently affects me. (PDtemp_01)*	.31	-	-
The COVID-19 pandemic will still affect me in five years. (PDtemp_02)*	.90	.92	.46
The COVID-19 pandemic will affect me for many years to come. (PDtemp_03)*	.82	.81	.42
**Social**			
The COVID-19 pandemic mainly affects people like me. (PDsoci_01)*	.38	.68	.43
The COVID-19 pandemic mainly affects my family and friends. (PDsoci_02)*	.30	.56	.37
The COVID-19 pandemic mainly affects other people. (PDsoci_03)	.42	-	-
**Hypothetical**			
The COVID-19 pandemic will most likely affect me. (PDhypo_01)*	.74	.74	.75
The COVID-19 pandemic is questionable to affect me. (PDhypo_02)	.73	.72	.69
The COVID-19 pandemic is unlikely to affect me. (PDhypo_03)	.70	.70	.66
Chi-square test (*degrees of freedom*)	303.982 (48)	46.478 (21)	316.645 (27)
Comparative Fit Index (CFI)	.79	.97	.67
Root Mean Square Error of Approximation (RMSEA)	.12	.06	.18
Standardized Root Mean Square Residual (SRMR)	.11	.04	.10
Akaike Information Criterion (AIC)	14914.904	10740.778	11036.805
Bayesian Information Criterion (BIC)	15033.888	10835.965	11108.196

*Note: * = Items were reversed due to the theoretical definition as distance and not closeness.*
