# Supplementary material for: Psychological distance towards COVID-19: Geographical and hypothetical distance predict attitudes and mediate knowledge
Source: Curr Psychol. 2021 Oct 31;42(10):8632–43. doi: 10.1007/s12144-021-02415-x (PMC8557103; doi:10.1007/s12144-021-02415-x)
Supplement: Supplementary file 3 — Factor analysis of attitudes (ESM 3) (PDF 104 kb) [file 12144_2021_2415_MOESM3_ESM.pdf]

Supplementary table 3

Results of the explorative factor analysis with the corresponding factor loadings (λ). The rotation method varimax was applied. No further settings were applied.

Item	factor loading (λ)		
	factor 1	factor 2	factor 3
**Affective**			
The measures to contain COVID-19 imposed from March onward have made my life easier. (att_pos_aff_01)	.43	**.52**	.23
The measures to contain COVID-19 imposed from March onward have pleased me. (att_pos_aff_02)	.23	**.69**	.13
The measures to contain COVID-19 imposed from March onward have dismayed me. (att_neg_aff_01)*	.04	**.75**	-.08
The measures to contain COVID-19 imposed from March onward have frustrated me. (att_neg_aff_02)*	.18	**.72**	.10
**Cognitive**			
The measures to contain COVID-19 imposed from March onwards were, I believe, appropriate. (att_pos_cog_01)	**.84**	.19	.16
The measures to contain COVID-19 imposed in March were, in my opinion, effective. (att_pos_cog_02)	**.85**	.06	.17
The measures to contain COVID-19 imposed in March were, in my opinion, disproportionate. (att_neg_cog_01)*	**.79**	.24	.14
The measures to contain COVID-19 imposed in March were, in my opinion, not far-reaching enough. (att_neg_cog_02)*	.10	-.39	-.35
**Behavioral**			
The measures to contain COVID-19, which were imposed from March on, I followed. (att_pos_beh_01)	.42	.17	**.64**
The measures to contain COVID-19, which were imposed from March on, have made me avoid the public. (att_pos_beh_02)	.50	-.02	**.64**
The measures to contain COVID-19, which were imposed in March, I ignored. (att_neg_beh_01)*	.40	.15	**.61**
The measures to contain COVID-19 imposed in March did not prevent me from going out into the public. (att_neg_beh_02)*	.01	.04	**.75**

Note. * = Items were reversed due to a negative formulation.
