# Supplementary material for: Psychological distance towards COVID-19: Geographical and hypothetical distance predict attitudes and mediate knowledge
Source: Curr Psychol. 2021 Oct 31;42(10):8632–43. doi: 10.1007/s12144-021-02415-x (PMC8557103; doi:10.1007/s12144-021-02415-x)
Supplement: Supplementary file 4 — Detailed results for moderation analysis (ESM 4) (PDF 114 kb) [file 12144_2021_2415_MOESM4_ESM.pdf]

Supplementary table 4
Regression results of the path model with standardized regression coefficients (β), standard error (SE), and 95% confidence interval (CI).

			95% CI	
Predictors	β	SE	LL	UL
**Warning-app (adj. R^2 = .10)**				
Affective attitudes	.09	.03	-.01	.09
Cognitive attitudes	.19**	.03	.04	.15
Geographical distance	.04	.03	-.04	.08
Temporal distance	.01	.02	-.04	.05
Social distance	.04	.02	-.03	.06
Hypothetical distance	-.18**	.03	-.14	-.03
**Behavioral attitudes (adj. R^2 = .33)**				
Affective attitudes	.13**	.04	.03	.17
Cognitive attitudes	.40***	.05	.23	.44
Geographical distance	-.01	.04	-.10	.07
Temporal distance	-.06	.04	-.11	.03
Social distance	.00	.04	-.07	.07
Hypothetical distance	-.16**	.04	-.21	-.04
**Affective attitudes (adj. R^2 = .06)**				
Geographical distance	-.09	.06	-.21	.02
Temporal distance	.06	.05	-.04	.15
Social distance	-.04	.05	-.14	-.07
Hypothetical distance	-.19**	.06	-.31	-.07
**Cognitive attitudes (adj. R^2 = .21)**				
Geographical distance	-.26***	.06	-.37	-.13
Temporal distance	-.02	.05	-.10	.07
Social distance	.04	.04	-.04	.11
Hypothetical distance	-.28***	.06	-.38	-.14

Note. LL = lower limit, UL = upper limit; * = $p < 0.05$, ** = $p < 0.01$, *** = $p < 0.001$, Model fit: $\chi 2$ (28) = 578.331, Comparative Fit Index (CFI) = 1.00, Root Mean Square Error of Approximation (RMSEA) = .00, Standardized Root Mean Square Residual (SRMR) = .00.
